# Combination of Preoperative Multimodal Image Fusion and Intraoperative Dyna CT in Percutaneous Balloon Compression of Trigeminal Ganglion for Primary Trigeminal Neuralgia: Experience in 24 Patients

**DOI:** 10.3389/fsurg.2022.895394

**Published:** 2022-05-09

**Authors:** Chang-chun Liao, Jia-yan Li, Kai-hua Wu, Zhi-heng Jian, Xin-feng YI, Zhi-jian Weng, Gang Chen

**Affiliations:** Department of Neurosurgery, Zhuhai People’s Hospital (Zhuhai Hospital Affifiliated With Jinan University, China), Zhuhai, China

**Keywords:** primary trigeminal neuralgia, percutaneous balloon compression, multimodal image fusion, DYNA CT, curative effect

## Abstract

**Objective:**

We retrospectively assessed the surgical results of PBC with preoperative multimodal image fusion and intraoperative Dyna Computed Tomography (CT) in 24 patients with primary trigeminal neuralgia (PTN) to explore a valuable aid for Percutaneous balloon compression (PBC).

**Methods:**

We studied the data of 24 patients with PTN. All patients underwent PBC and were assessed with preoperative multimodal image fusion [computed tomography (CT) and magnetic resonance imaging (MRI)] and intraoperative Dyna CT in the Department of Neurosurgery of Zhuhai People’s Hospital between October 2020 and September 2021. Multimodal image fusion—three-dimensional (3D) reconstruction of CT and MRI data—was performed using 3D-Slicer software, and preoperative evaluation was performed according to the results of image fusion. Dyna CT was used to dynamically observe the position and shape of the metallic hollow introducer and Fogarty catheter and balloon during the operation to guide the operation in real time. We performed follow-up assessments each month and summarized the clinical characteristics, surgical effects, and complications in all patients.

**Results:**

Surgery was successful for all patients; the patients reported immediate pain relief. Surgical complications included facial numbness in 24 patients (100%), mild masseter weakness in three (12.5%), herpes zoster in three (12.5%), and balloon rupture in one (4.2%). None of the patients had serious surgical complications. The mean follow-up time was 9.6 ± 2.7 months. During the follow-up period, 22 patients (91.7%) experienced no recurrence of pain, and two patients (8.3%) experienced recurrence of pain, of which one underwent secondary PBC surgery.

**Conclusions:**

Preoperative multimodal image reconstruction can help fully evaluate PBC surgery, clarify the etiology, and predict the volume of contrast medium required during the operation. It provided important assistance for PBC treatment of trigeminal neuralgia patients when preoperative multimodal image fusion is combined with intraoperative Dyna CT.

## Introduction

Primary trigeminal neuralgia (PTN) is a chronic neuropathic pain disorder characterized by spontaneous and elicited paroxysms of electric shock-like or stabbing pain in a region of the face (1–3). The pain of trigeminal neuralgia may be triggered by common movements of the face, such as talking, washing the face, chewing, brushing the teeth, or eating. Poor quality of life and suicide (in severe cases) have been attributed to the disorder (1, 4–7). In 1983, Mullan & Lichtor first described the percutaneous microballoon compression (PBC) technique for trigeminal neuralgia (8), which has been used since. Although it is generally accepted that microvascular decompression is the preferred surgical treatment for PTN (success rate 62%–89%) (9), PBC remains a popular treatment, especially for some elderly patients or patients with postoperative recurrence (10). However, facial numbness, masseter weakness, herpes zoster and other complications often occur after PBC (11, 12), and severe complications, such as foramen ovale (FO) puncture failure, and even rupture of the internal carotid artery may occur. Therefore, correct puncture and successful balloon compression are crucial.

At present, preoperative imaging techniques, such as thin-slice CT, three-dimensional (3D) time-of-flight (TOF) MR angiography (MRA) (3D-TOF-MRA), and 3D TSE DRIVE, are often used for preoperative evaluation (13, 14), but these involve the assessment of two dimensional (2D) images. In this study, multi-modal image fusion technology was adopted. We processed digital imaging and communications in medicine (DICOM) data to preoperatively reconstruct 3D images using the software 3D-Slicer (15–17), and then observed the relationship between nerves and peripheral blood vessels more intuitively and accurately, measured the shape and volume of the Meckel cavity, observed the shape of the FO, and simulated the puncture of the FO. During the operation, Dyna CT (18, 19) was used to scan and make 3D model of the skull, and FO puncture was performed with the assistance of dynamic X-ray fluoroscopy and 3D model navigation. Using Dyna CT, the position and shape of metallic hollow introducer, catheter and balloon were observed in real time, and could be adjusted at any time. We attempted to improve the safety and effectiveness of PBC through the application of preoperative multimodal image fusion and intraoperative Dyna CT.

## Materials

### Patients

The pre-and postoperative clinical data (obtained from medical records) of 24 patients treated with PBC for PTN between October 2020 and September 2021 were reviewed. All our patients were treated in the Department of Neurosurgery of Zhuhai People’s Hospital. Our inclusion criteria were (1) typical PTN; (2) age >55 years; (3) poor or no response to drug treatment; (4) no remission or recurrence after previous microvascular decompression (MVD). We excluded patients with secondary trigeminal neuralgia and those who could not be followed up for more than 3 months after surgery. All studies were conducted with written informed consent of patients.

A total of 24 patients were included in this research group, including 8 men and 16 women aged 52–82 years (average age 67.5 ± 8.5 years); the duration since PTN onset was 1.5–16 (6.0 ± 4.1) years. Seven patients experienced left trigeminal neuralgia and 17 patients experienced right side symptoms. One patient had a five-year recurrence after MVD. One patient underwent a second PBC operation in this study due to postoperative recurrence of PBC (Table 1).

**Table 1:** Summary table of patient demographics (*n* = 24).

Characteristic	Value
Sex, *n*	
Male	8
Female	16
Age, years, range (mean ± SD)	67.5 ± 8.5
Side affected, *n*	
Left	7
Right	17
Time since TN diagnosis, months, range (mean ± SD)	6.0 ± 4.1
TN pain distributions	
Single-branch disease, *n*	
V2 only	3
V3 only	7
Multi-branch disease, *n*	
V1/2	2
V2/3	9
V1/2/3	3
Prior surgical treatment, *n*	
Treated with PBC	1
Treated with MVD	1

### Equipment

(1) MRI scanner: PhilipsIngenia CX 3.0 T MRI scanner; the thickness of the scanning layer used was 1.0 mm, the MR scanning sequence included T1WI 3D TFE, 3D TSE DRIVE, 3D TOF MRA, 3D-FLAIR-Real-Meniere (2). CT scanner: United Imaging UCT 780; the thickness of the scanning layer used was 0.63 mm (3). Digital subtraction angiography (DSA) and Dyna CT: Siemens Artis Q Zeego (4). Fogarty catheter (FC): from Shenzhen SYM Medical Instrument Co. Ltd (4). Size of fogarty balloon: 1 mL/2 mL. We used DICOM data for this research.

## Methods

### Preoperative Multimodal Image Fusion and Evaluation

**Figure 1:**
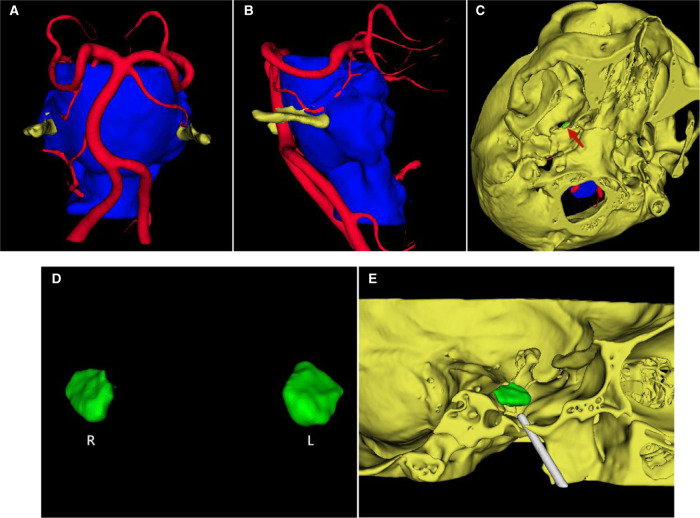
Multimodal image fusion and 3D reconstruction. (**A**) The multimodal image fusion and 3D reconstruction of brain stem, cranial nerves, blood vessels and skull by 3D-Slicer, showing the relationship between the left trigeminal nerve and the left superior cerebellar artery (front view). (**B**) Left view. (**C**) Image made of the FO morphology by 3D reconstruction of the skull (view below). (**D**) The bilateral Meckel cavities. (**E**) Simulation of FO puncture.

The DICOM data of the 24 patients were obtained from the hospital imaging department, and were used in multimodal image fusion and 3D reconstruction of the brain stem, cranial nerves, blood vessels and skull using the software 3D-Slicer (Figure 1), and then evaluated based on three criteria: (1) whether there were vessels pressing the trigeminal nerve which could be responsible for the neuralgia; (2) FO morphology: whether there was FO stenosis or a sharp bone crest; (3) the volume of bilateral Meckel cavities, and the volume of contrast medium required for full balloon inflation during surgery was estimated.

### Surgical Procedure

**Figure 2:**
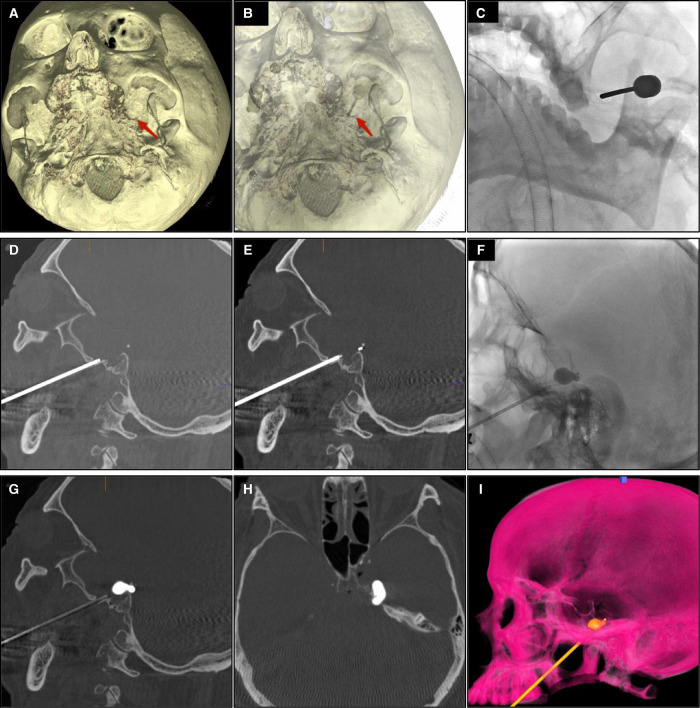
(**A**) Intraoperative 3D reconstruction of the skull immediately after Dyna CT scan. (**B**) The FO puncture was performed under the 3D skull image. (**C**) The DSA dynamic fluoroscopy. (**D**) Dyna CT scan was performed to adjust the direction and depth of the metallic HI to the correct position. (**E**) The distal mark of the FC was located at the apex of the Meckel chamber. (**F**) The balloon was fully inflated and pear-shaped. (**G**) Balloon morphology under Dyna CT (sagittal view). (**H**) Balloon morphology under Dyna CT (axial view). (**I**) Balloon morphology in 3D rendering.

(1) Anesthesia and head position: surgery was performed under brief general anesthesia with intratracheal intubation. The patient was in the supine position with the head tilted back 10–15°, and the head was fixed to avoid intraoperative head movement.
(2) Dyna CT scan of the head was performed to obtain 3D images of the patient’s skull and FO (Figure 2A).
(3) FO puncture: The balloon attached to the FC balloon was filled with contrast medium in order to check its patency and to allow air to escape. The skin puncture point was 2.5 cm lateral to the angle lip and the FO puncture was performed under the guidance of 3D skull image (Figure 2B) and DSA dynamic fluoroscopy (Figure 2C).
(4) Needle depth was adjusted and confirmed. Sagittal X-rays were taken to show the metallic hollow introducer (HI) in close contact with the posterior extremity of the horizontal plate of the palatine bone and at the level of the FO, not exceeding the clivus and the superior edge of the petrous bone. Then a Dyna CT scan was performed to adjust the direction and depth of the metallic HI to the correct position (Figure 2D).
(5) FC placement: The HI was withdrawn while the FC was gently pushed up 34 mm toward the Gasser ganglion, until the distal mark of the FC was located at the apex of the Meckel chamber (Figure 2E) (5). The balloon was inflated by injecting the proper amount of contrast medium into the balloon slowly, with the goal of fully inflating the balloon into a pear shape (Figure 2F).
(6) Continuous balloon compression was then performed for 2–3 min, during which time Dyna CT was used to scan the head (Figure 2G–I).
(7) Anabiosis was performed immediately after the operation and the relief of facial pain was evaluated. (7) Transient bradycardia may occur during FO puncture and atropine may be used if necessary. Elevation of the blood pressure can occur concurrently with the onset of bradycardia. Patients were generally discharged within 48 h of surgery.

### Assessment Criteria

(1) Efficacy evaluation: Grade A: the pain disappeared completely without oral analgesics; Grade B: partial relief, postoperative pain still occurred, but the intensity and frequency of pain were significantly improved; Grade C: no obvious relief or no relief of facial pain after surgery.
(2) Complications included facial hypoesthesia, masseter weakness, herpes zoster, keratitis, diplopia, hearing loss, meningitis, balloon rupture.
(3) Follow-up: telephone or outpatient follow-up was conducted every month for 3–12 months to determine whether there was pain recurrence or any complications. In this study, 24 patients were followed up regularly after surgery.
(4) Statistical analysis: Paired sample T test were used to assess differences in Volume of the bilateral MC using IBM SPSS Version 22.0 (IBM Corp., Armonk, New York, USA). *P* < 0.05 was considered statistically significant.

## Results

According to preoperative multimodal image fusion, 20 (83.3%) of the 24 patients were considered to have vascular compression, and 4 (16.7%) were not found to have significant vascular compression. The Meckel cavity volume was 0.36 ± 0.14 mL (range: 0.18–0.68 mL) on the affected side and 0.37 ± 0.14 mL (range: 0.13–0.72 mL) on the healthy side, and there was no statistically significant difference between the two (*t* = 0.221, *p* = 0.827). The Meckel cavity volume on the left was 0.35 ± 0.14 mL (range: 0.18–0.72 mL), and that on the right was 0.37 ± 0.14 mL (range: 0.13–0.68 mL), and there was no statistically significant difference between the two (*t* = 1.244, *p* = 0.226) (Table 2).

**Table 2:** Mean Volume of MC (*n* = 24).

	Volume of MC (mL)	*t*	*p*
Affected side	0.36 ± 0.14 (0.18–0.68)	0.221	0.827
Unaffected side	0.37 ± 0.14 (0.13–0.72)		
Left	0.35 ± 0.14 (0.18–0.72)	1.244	0.226
Right	0.37 ± 0.14 (0.13–0.68)		

**Figure 3:**
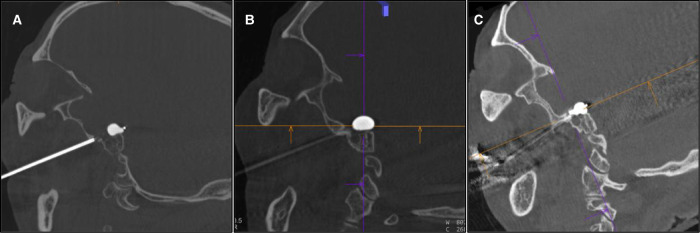
Different balloon morphology (**A**) Pear shape. (**B**) Spherical. (**C**) Irregular shape.

With intraoperative Dyna CT assistance, all 24 patients successfully completed surgery and were scored with Grade A relief of the pain (100%). The volume of contrast medium required for full balloon inflation was 0.56 ± 0.21 mL (range: 0.35–1.4 mL), which was 1.67 ± 0.54 (0.85–2.8) times the volume of MC. Among the 24 operations, 21 (87.5%) balloon shapes were typical pear-shaped, 2 were atypical pear-shaped, 1 was spherical, and 1 was irregular (Figure 3). Intraoperative balloon rupture occurred once, and no serious complications, such as internal carotid artery rupture or intracranial hemorrhage, occurred. Postoperative facial hypoesthesia was found in all patients, all within the acceptable range.

The mean follow-up time was 9.6 ± 2.7 months, postoperative follow-up time between 6 and 15 months. During follow-up, 22 patients (91.7%) had no recurrence of pain and one patient, in whom the balloon shape was spherical, relapsed 2 weeks later. This patient underwent a second PBC operation in our hospital. After the second operation, the patient had no further pain. Another patient relapsed 1 week after surgery, and their pain could be controlled by intermittent administration of carbamazepine. Three patients (12.5%) experienced herpes zoster infection within 1 week following surgery, three patients (12.5%) had masseter weakness after surgery, and none of the patients had serious complications.

### A Notable Case

**Figure 4:**
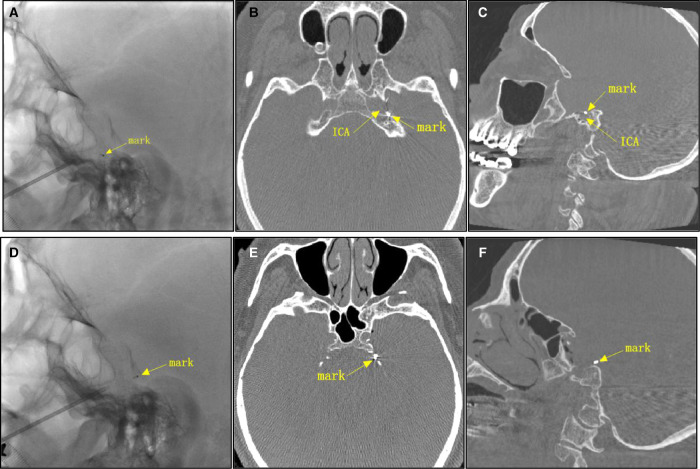
A notable case.The yellow arrow shows the distal mark of the FC.

The patient shown in Figure 4 was a 69-year-old patient with left trigeminal neuralgia. The research group were unable to push the FC toward the Gasser ganglion, so the FC was unable to reach the ideal position (Figure 4A). Dyna CT showed that the FC was pushed in the direction of internal carotid artery and was stuck (Figure 4B,C). If the FC were pushed forcibly at this time, the internal carotid artery may have ruptured, resulting in serious consequences. The research group adjusted the direction of FC according to CT results in time and the FC was positioned appropriately (Figure 4D–F).

## Discussion

In recent years, preoperative multimodal image fusion is being widely applied in the field of neurosurgery (20–24), especially for diseases such as brain tumor, trigeminal neuralgia, and facial spasm. The use of preoperative multimodal image fusion technology can more intuitively display skin, skull, brain tissue, nerve fiber bundle, tumor, and blood vessel to the surgical team.

In this study, we also conducted preoperative multimodal image fusion in other patients with trigeminal neuralgia and facial spasm. Based on the modal auxiliary image fusion evaluation treatment, we were able to better guide the practices in surgical procedures, particularly during the MVD operations. Preoperative vascular multimodal image fusion could help identify responsible vessels and understand the spatial relationship between nerves and blood vessels. Thus, the risks associated with the operation could be reduced, and the recovery rate of operation could be improved.

In this study, the use of preoperative multimodal image fusion technology was focused on preoperative observations of FO morphology to understand the presence of FO stenosis or sharp bone crest, to mitigate the risk of intraoperative FO puncture failure or balloon rupture caused by FO stenosis or sharp bone crest.

In addition, the Meckel cavity was reconstructed to observe its morphology, and the size of the of the Meckel cavity on the affected side was calculated. The study found that the actual intraoperative contrast dose used during surgery was 1.67 ± 0.54 (0.85–2.8) times of the preoperative calculation of the Meckel cavity size, which provided a good reference for the neurosurgeon.

Most commonly, a 1 mL volume balloon was used in the operations, but in the case of a larger Meckel cavity volume, a larger size balloon was needed, as otherwise the balloon was prone to rupture or other risks. It was helpful to be able to choose the appropriate balloon catheter before surgery.

Using multimodal image fusion technology, we collected the size of bilateral Meckel cavities of patients. There was no statistically significant difference in the volume of Meckel cavities between the affected side and the healthy side, indicating that the volume of Meckel cavities may have no relationship with the occurrence of trigeminal neuralgia. By comparing bilateral Meckel cavities, the research group also found that there was no statistically significant difference between the volume of the left and right Meckel cavities. However, Zhang et al. (25) have pointed out that trigeminal neuralgia may be related to the morphology and flatness of the Meckel cavity. This may be studied further in the future.

All previous PBC surgeries were completed using a C-arm machine, and the surgeons needed to perform FO punctures repeatedly under the anterior and posterior FO and to use lateral image fluoroscopy. Occasionally, this resulted in puncture failure, puncture error, and internal carotid artery injury. According to the report of B Abdennebi (26), 82 (9.1%) of 901 patients underwent surgery failed due to various causes, including the impossibility to cross the FO and Atypical balloon shape. In this study, Dyna CT was used to assist PBC treatment, and 3D images were used for real-time navigation to make FO puncture more intuitive. When difficulties are encountered in the puncture process, Dyna CT scan during the operation can be used to find the cause of puncture failure and adjust the direction of the puncture needle in a timely manner.

Improper placement of the balloon catheter may occur in PBC surgery, and lead to inadequate expansion of the balloon. Unsatisfactory shape of the balloon and incorrect compression position ultimately result in poor surgical outcomes, such as a high rate of recurrence. While it was difficult to locate the catheter specifically with the conventional C-arm lateral image perspective, we were able to solve this problem well with the application of intraoperative Dyna CT. For example, in Figure 4, we were able to accurately observe the position of balloon catheter using the intraoperative Dyna CT, thus purposefully adjusting the placement direction of the balloon catheter. If the distal end of the balloon catheter reached the entrance of Meckel cavity, the balloon would be standard pear-shaped after full expansion, and the surgical effect was satisfactory.

In follow-up, spherical balloon dilation occurred in the first operation and recurred 2 weeks after surgery. In the second operation, the balloon catheter position was adjusted to ensure that the balloon dilated in a standard pear-shaped shape. Therefore, we concluded that the main reason for recurrence of patients after the first operation was related to the position of catheter and balloon shape. However, the balloon shape of another patient who relapsed was also standard pear-shaped, but the facial pain also recurred 2 weeks after surgery, which may be a result related to the compression time and balloon pressure.

Preoperative multimodal image fusion technology evaluated the preoperative treatment of PTN and predicted the intraoperative situation, but it provided no-real-time intraoperative guidance. The application of intraoperative Dyna CT made up for this disadvantage, and the combined application of the two provided full guidance for the treatment of primary trigeminal nerve PBC.

However, there were several issues with this study—(1) The fluoroscopy time and radiation dose during PMC would increase due to the application of intraoperative Dyna CT, but it was appropriate to use dyna-CT in these cases for it faciliated puncture of the FO when difficulty occurred and provided important data for improvement of operation techniques (2) the number of cases was too small to provide strong statistical evidence (3) accurate balloon pressure measurement was not performed during balloon expansion—which may have affected the reliability of research data to some extent. In further studies, we intend to study more cases and perform detailed quantitative analyses to obtain more reliable data.

## Conclusions

Preoperative multimodal image reconstruction can fully evaluate PBC surgery, clarify the etiology, and predict the volume of contrast medium required during the operation. It provided important assistance for PBC treatment of trigeminal neuralgia patients when preoperative multimodal image fusion is combined with intraoperative Dyna CT.

## Data Availability

The original contributions presented in the study are included in the article/supplementary material, further inquiries can be directed to the corresponding author/s.

## References

[1] Cruccu G Di Stefano G Truini A. Trigeminal neuralgia. N Engl J Med. (2020) 383:754–62. 10.1056/NEJMra1914484 32813951

[2] Maarbjerg S Di Stefano G Bendtsen L Cruccu G. Trigeminal neuralgia - diagnosis and treatment. Cephalalgia. (2017) 37:648–57. 10.1177/0333102416687280 28076964

[3] Bendtsen L Zakrzewska JM Heinskou TB Hodaie M Leal PR Nurmikko T Advances in diagnosis, classification, pathophysiology, and management of trigeminal neuralgia. Lancet Neurol. (2020) 19:784–96. 10.1016/S1474-4422(20)30233-7 32822636

[4] Finnerup NB Kuner R Jensen TS. Neuropathic pain: from mechanisms to treatment. Physiol Rev. (2021) 101:259–301. 10.1152/physrev.00045.2019 32584191

[5] Tepper SJ. Cranial neuralgias. Continuum (Minneap Minn). (2018) 24(4, Headache):1157–78. 10.1212/CON.0000000000000637 30074554

[6] Zakrzewska JM Wu J Mon-Williams M Phillips N Pavitt SH. Evaluating the impact of trigeminal neuralgia. Pain. (2017) 158:1166–74. 10.1097/j.pain.0000000000000853 28114183

[7] Melek LN Smith JG Karamat A Renton T. Comparison of the neuropathic pain symptoms and psychosocial impacts of trigeminal neuralgia and painful posttraumatic trigeminal neuropathy. J Oral Facial Pain Headache. (2019) 33:77–88. 10.11607/ofph.2157 30703173

[8] Mullan S Lichtor T. Percutaneous microcompression of the trigeminal ganglion for trigeminal neuralgia. J Neurosurg. (1983) 59:1007–12. 10.3171/jns.1983.59.6.1007 6631493

[9] Bendtsen L Zakrzewska JM Abbott J Braschinsky M Di Stefano G Donnet A European Academy of Neurology guideline on trigeminal neuralgia. Eur J Neurol. (2019) 26:831–49. 10.1111/ene.13950 30860637

[10] Ying X Wang H Deng SH Chen Y Zhang J Yu W. Long-term outcome of percutaneous balloon compression for trigeminal neuralgia patients elder than 80 years. Med (Baltim). (2017) 96:e8199. 10.1097/MD.0000000000008199 PMC5626327 28953684

[11] Xu R Xie ME Jackson CM. Trigeminal neuralgia: current approaches and emerging interventions. J Pain Res. (2021) 14:3437–63. 10.2147/JPR.S331036 34764686 PMC8572857

[12] Li MW Jiang XF Niu CS. Efficacy of and risk factors for percutaneous balloon compression for trigeminal neuralgia in elderly patients. Br J Neurosurg. (2021) 35:280–4. 10.1080/02688697.2020.1787341 32619112

[13] Brînzeu A Drogba L Sindou M. Reliability of MRI for predicting characteristics of neurovascular conflicts in trigeminal neuralgia: implications for surgical decision making. J Neurol Surg. (2018) 130:611–21. 10.3171/2017.8.JNS171222 29624148

[14] Zeng C Zhang C Li YH Feng X Zhang MJ Xiao RH Recent advances of magnetic resonance neuroimaging in trigeminal neuralgia. Curr Pain Headache Rep. (2021) 25:37. 10.1007/s11916-021-00957-0 33821366

[15] Han KW Zhang DF Chen JG Hou LJ. Presurgical visualization of the neurovascular relationship in trigeminal neuralgia with 3D modeling using free Slicer software. Acta Neurochir (Wien). (2016) 158:2195–201. 10.1007/s00701-016-2936-8 27543280

[16] Cheng J Meng J Liu W Zhang H Hui X Lei D. Primary trigeminal neuralgia is associated with posterior fossa crowdedness: a prospective case-control study. J Clin Neurosci. (2018) 47:89–92. 10.1016/j.jocn.2017.10.032 29066228

[17] Bruns N. 3D Slicer: universal 3D visualization software. Unfallchirurg. (2019) 122:662–3. 10.1007/s00113-019-0654-4 31286152

[18] Huang B Yao M Chen Q Du X Li Z Xie K Efficacy and safety of awake computed tomography-guided percutaneous balloon compression of trigeminal ganglion for trigeminal neuralgia. Pain Med. (2021) 22:2700–7. 10.1093/pm/pnab228 34320638

[19] Xiao X Wei Z Ren H Sun H Luo F. Comparison of effectiveness and safety between intraoperative 3D-CT-guided and C-arm-guided percutaneous balloon compression for idiopathic trigeminal neuralgia: a multi-center retrospective study. Pain Res Manag. (2021) 2021:9306532. 10.1155/2021/9306532 34194588 PMC8203368

[20] Huang B Yang F Yin M Mo X Zhong C. A review of multimodal medical image fusion Techniques. Techniques. Comput Math Methods Med. (2020) 2020:8279342. 10.1155/2020/8279342 32377226 PMC7195632

[21] Yang R Li QX Mao C Peng X Wang Y Guo YX Multimodal image fusion technology for diagnosis and treatment of the skull base-infratemporal tumors. Beijing Da Xue Bao Yi Xue Ban. (2019) 51:53–8. 10.19723/j.issn.1671-167X.2019.01.010 PMC7433559 30773544

[22] Yadav SP Yadav S. Image fusion using hybrid methods in multimodality medical images. Med Biol Eng Comput. (2020) 58:669–87. 10.1007/s11517-020-02136-6 31993885

[23] Piccinelli M. Multimodality image fusion, moving forward. J Nucl Cardiol. (2020) 27:973–5. 10.1007/s12350-019-01607-0 30693427 PMC6661216

[24] Zhang P Wang G Sun Z Lv X Guo Y Wang J Application of multimodal image fusion to precisely localize small intramedullary spinal cord tumors. World Neurosurg. (2018) 118:246–9. 10.1016/j.wneu.2018.07.034 30031956

[25] Lin J Zhang Y Li W Yan J Ke Y. Flatness of the Meckel cave may cause primary trigeminal neuralgia: a radiomics-based study. J Headache Pain. (2021) 22:104. 10.1186/s10194-021-01317-4 34479476 PMC8414677

[26] Abdennebi B Guenane L. Technical considerations and outcome assessment in retrogasserian balloon compression for treatment of trigeminal neuralgia. series of 901 patients. Surgical Neurology International. (2014) 5(1):118. 10.4103/2152-7806.137838 25101213 PMC4123256

